# Deconvolution Enhancement Keypoint Network for Efficient Fish Fry Counting

**DOI:** 10.3390/ani14101490

**Published:** 2024-05-17

**Authors:** Ximing Li, Zhicai Liang, Yitao Zhuang, Zhe Wang, Huan Zhang, Yuefang Gao, Yubin Guo

**Affiliations:** 1 College of Mathematics and Informatics, South China Agricultural University, Guangzhou 510642, China; liximing@scau.edu.cn (X.L.); wangzhe@stu.scau.edu.cn (Z.W.); 2 College of Foreign Studies, South China Agricultural University, Guangzhou 510642, China

**Keywords:** fish fry counting, keypoint, deconvolution, heatmap

## Abstract

**Simple Summary:**

Fish fry counting finds applications in various scenarios, including fish fry trading and the management of breeding densities. However, current computer-based methods struggle to accurately and effectively count large numbers of fish fry and locate them. To address these challenges, this study proposes a fish fry counting method based on a single keypoint as a feature. Additionally, a large-scale fish fry dataset, FishFry-2023, has been constructed, which includes universal point annotations for use. The number of fry in a single image ranges from 204 to 1935, ensuring an effective evaluation of the proposed method. Our approach was trained and tested on FishFry-2023, with experimental results demonstrating an average counting accuracy of 98.59%, exhibiting higher counting precision compared to the density map algorithms now available.

**Abstract:**

Fish fry counting has been vital in fish farming, but current computer-based methods are not feasible enough to accurately and efficiently calculate large number of fry in a single count due to severe occlusion, dense distribution and the small size of fish fry. To address this problem, we propose the deconvolution enhancement keypoint network (DEKNet), a method for fish fry counting that features a single-keypoint approach. This novel approach models the fish fry as a point located in the central part of the fish head, laying the foundation for our innovative counting strategy. To be specific, first, a fish fry feature extractor (FFE) characterized by parallel dual branches is designed for high-resolution representation. Next, two identical deconvolution modules (TDMs) are added to the generation head for a high-quality and high-resolution keypoint heatmap with the same resolution size as the input image, thus facilitating the precise counting of fish fry. Then, the local peak value of the heatmap is obtained as the keypoint of the fish fry, so the number of these keypoints with coordinate information equals the number of fry, and the coordinates of the keypoint can be used to locate the fry. Finally, FishFry-2023, a large-scale fish fry dataset, is constructed to evaluate the effectiveness of the method proposed by us. Experimental results show that an accuracy rate of 98.59% was accomplished in fish fry counting. Furthermore, DEKNet achieved a high degree of accuracy on the Penaeus dataset (98.51%) and an MAE of 13.32 on a public dataset known as Adipocyte Cells. The research outcomes reveal that DEKNet has superior comprehensive performance in counting accuracy, the number of parameters and computational effort.

## 1. Introduction

Counting, which is defined as estimating the number of objects in the target area to obtain precise information and perform timely control operations, has been widely applied in many different fields [1,2]. For example, Han et al. [3] figured out the total number of individual pedestrians using a density estimation method. Akçay et al. [4] employed object detection models utilizing deep neural networks to assist the automatic process of bird counting. Fan et al. [5] counted fish fry using computer vision and a multi-class least squares support vector machine. Fish fry counting is an important task in the aquaculture industry. For one thing, fish fry counting is an essential part of aquatic product trading. Culturing fish fry requires particular climate conditions, light conditions and water quality, so most aquatic farms need to purchase fish fry from those that specialize in hatching fry, which inevitably involves fish fry counting. For another, fish fry counting can also provide guidance on assessing the survival rate, regulating breeding density, and monitoring transportation sales [6]. Therefore, an automated, efficient and accurate counting method is pivotal in the development of fish farming.

In general, fish fry counting methods include manual counting, physical shunt equipment counting, image processing counting and deep learning-based counting. Manual counting is time consuming, labor intensive, and costly, and its accuracy is likely to be affected by human fatigue and other subjective factors during the counting process. As for physical shunt equipment counting, it is not desirable as it may cause damage to fish, harming fish welfare.

Currently, researchers are mainly exploring image processing counting and deep learning-based counting. For example, Hernández-Ontiveros et al. [7] implemented fish counting through digital image processing with an accuracy rate of 96.64%. Aliyu et al. [8] first segmented the images by applying three methods: (1) image binarization using Otsu thresholding; (2) morphological operations using fill hole, dilation, and opening operations; (3) boundary segmentation using edge detection. Then, the chain code algorithm and Fourier descriptor were leveraged to extract boundary features. Finally, the boundary features were input to the neural network to identify and count the catfish. The proposed counting algorithm performed well, demonstrating 100% accuracy. Zhang et al. [6] used binarization, dilation, and corrosion methods to extract the details of the fish fry. The thinning and the connected area algorithms were then independently adopted to count the fish fry within the image. These research efforts are based on traditional image processing methods to conduct fish counting. Nevertheless, they have certain limitations. Although it is possible to roughly estimate the number of objects in the image, they cannot utilize more abstract semantic features and background information in the image, and the processing effect is disappointing in case of occlusion, congestion, high density, etc.

Zhang et al. [9] first used traditional image processing methods to separate different density grading, then used local regression to calculate the number of fry in different density grading, and finally added the number of fry in multiple density grading. This method delivered an accuracy rate of 96.07%. Zhao et al. [10] used density map regression to count fish fry by adding transposed layers and concentrated–comprehensive convolution modules to the generation head so that the resolution of the feature map is restored and the computational overhead is reduced. The proposed method achieved a mean absolute error of 4.10. Yu et al. [11] added an attention module to three parallel convolutional networks to extract feature maps, and finally used the density map estimation module to estimate the distribution and the number of fish in the image, with an accuracy rate of approximately 97.12% attained. Despite the high precision in counting fish with high-density levels, these methods based on deep learning fail to pinpoint the exact location of the fish. Therefore, the trading parties cannot be convinced in an intuitive way.

Deep learning technology marks a breakthrough in automatic counting methods in smart aquaculture. In addition to the density map regression method, some scholars also used the object detection algorithm to fulfill the counting function. Ditria et al. [12] selected Mask R-CNN for fish counting, and the proposed model achieved an accuracy up to 93.4%. Allken et al. [13] used RetinaNet to count fish, combining real images and synthetic images during the training phase. Its counting precision was 84.5%, indicating that there was still room for improvement. Jalal et al. [14] integrated optical flow and Gaussian mixture models with a YOLO deep neural network for detecting fish in underwater scenarios. Their proposed algorithm achieved an F-score of 95.47%. Cai et al. [15] performed fish detection and counting by extracting image features of fish with the YOLOv3 model, but its average counting precision was less than 80%. Lei et al. [16] proposed an improved detection method, termed as YOLOv7-waterbird, for real-time video surveillance devices to identify attention regions and perform waterbird monitoring tasks. Nevertheless, the mean average precision (mAP) value achieved by YOLOv7-waterbird was 67.3%. In addition to the unsatisfactory counting precision in previous studies, two more problems still need to be addressed; first of all, as the size of the fish fry is very small, methods proposed in these experiments to count the relatively large fish may not be applicable. Moreover, the detection-then-counting algorithms used in previous research may suffer from low counting efficiency and accuracy. For one thing, they involve post-processing, namely non-maximum suppression, in which the duplicate detection of the same instance should be eliminated by calculating the bounding box IoU [17]. For another, they are prone to treating multiple objects as a single one when these objects are relatively close to each other.

In recent years, the keypoint has been harnessed to detect objects and some scholars have used keypoints to achieve human pose estimation. Zhou et al. [17] modeled an object as a single point by first using keypoint estimation to find the center point, and then returning to other object properties such as size and orientation. Liu et al. [18] added an attention mechanism to the CenterNet backbone to improve the feature expression ability. Then, a circle representation was introduced to optimize the detector for detecting tomatoes. Although its precision reached 98.16%, the number of tomatoes in one image was not large. Chen et al. [19] designed a deep neural network to detect five keypoints in pig body parts, then correlated the keypoints to identify individual pigs, and finally designed a tracking algorithm to complete pig counting in the video. Its experimental results show that the highest accuracy could reach 99.2%. Due to the large size of the pig, five keypoints can be used concurrently to determine a pig, but further grouping is required to associate the five keypoints with the same target, which, to a certain extent, reduces the efficiency of the model.

**Figure 1:**
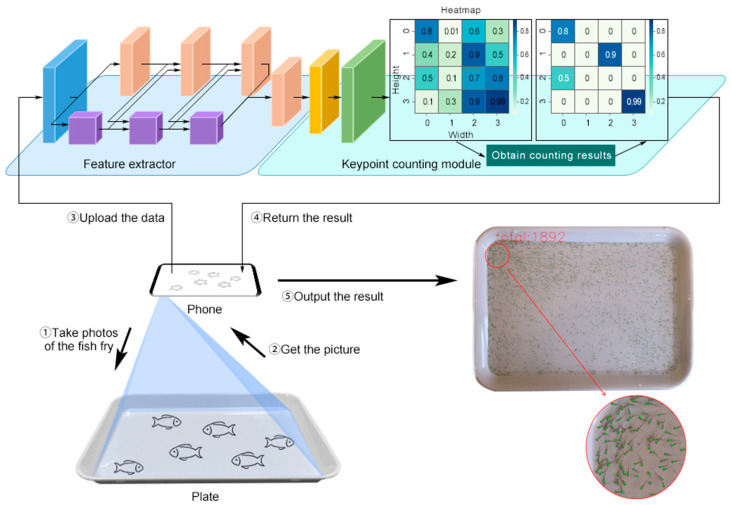
The pipeline of fish fry counting. A smartphone is first used to collect data, and the feature extractor is then used to extract high-resolution representation. Finally, the keypoint-counting module generates the counting results and locations of fish fry according to the heatmap.

Although these studies have yielded satisfying results, fish fry counting is still a very challenging task because of the severe occlusion, dense distribution and small size of fish fry aged 5–10 days. Besides, one single prediction to quickly and accurately calculate the maximum number possible of fish fry is valuable for commercial applications. Furthermore, some methods mentioned above fail to obtain the locations of fish fry, which can be utilized in subsequent higher-level fish fry analysis tasks, such as movement monitoring and anomaly detection. Therefore, we propose a deconvolution enhancement keypoint network (DEKNet), an automatic counting method that uses only one keypoint for fish fry counting. This is an end-to-end model which requires neither grouping nor post-processing. When using DEKNet for fry counting, there is no need to regress other properties of the object, and only counting the target is needed. The quantities and the locations of fish fry can be gained by obtaining the local peaks of the heatmap generated by DEKNet as the keypoint. The pipeline of fish fry counting is illustrated in Figure 1. First, the image is acquired with a smartphone and transmitted to DEKNet. Next, the heatmap is generated by DEKNet via a series of convolution operations. Finally, the local peak is obtained from the heatmap as the keypoint of the target, and the number of keypoints is counted, which equals to the number of fish fry.

DEKNet stands out with the following three characteristics:

1. High-resolution heatmap. Generating a high-resolution heatmap with superior quality is crucial for precisely locating the keypoints of small objects. However, a high-resolution heatmap is less investigated. Most methods mainly focus on grouping keypoints and simply using a single resolution of a feature map that is 1/4 of the input image resolution to predict the heatmap of keypoints. Coordinate regression based on the heatmap produces quantization errors, as the coordinates have their precision tied to the resolution in the heatmap [20]. For example, assume the input image size is 1024 × 1024, and one of the keypoints is located at 516 × 516. The resulting output heatmap would be 256 × 256, which is one-fourth the size of the original image. Consequently, due to downsampling, the keypoint would be positioned at 128 × 128. However, there might remain an error of 3 pixels (calculated as 516 − 128 × 4 = 3 pixels), even if the heatmap is restored without any discrepancies. Therefore, in order to handle quantization issues, two deconvolution (transposed convolution) modules are added to the generation head in DEKNet in order to generate a high-quality and high-resolution heatmap with the same resolution as the input image. Two factors made us select deconvolution. One reason is that deconvolution can be used in sequence at the end of a network to efficiently increase feature map resolution [21]. The other reason is that Xiao et al. [22] demonstrated that deconvolution can generate high-quality feature maps for heatmap prediction.
2. High-resolution representation. High-resolution representation is conducive to identifying the object. In the whole process, the two parallel branches of the feature extraction module simply perform feature extraction synchronous with feature fusion, but the resolution of their respective feature map remains unchanged, where the resolution of the feature map in the first branch is as high as 1/4 of the original image, while the resolution of the feature map in the second branch is as high as 1/8 of the original image. The feature extraction module ultimately outputs the feature map acquired in the first branch. As a result, DEKNet can obtain high-resolution representations throughout the feature extraction process.
3. Maximum efficiency. DEKNet is an end-to-end model that regresses keypoints through the heatmap. Aiming to achieve precise counting, DEKNet uses a single keypoint to fulfill the counting function and obtain the locations of fish fry without regressing other properties of the object. In addition, DEKNet only utilizes one keypoint for counting, which means that there is no need to group different types of keypoints, nor does it involve post-processing, thus greatly reducing the computational effort of the model.

Therefore, the main contributions of this pager are summarized below:

1. An automatic fry counting method (DEKNet) using one keypoint is proposed, which can effectively count the number of fish fry despite overlap and obscurity. The approach not only achieves state-of-the-art counting accuracy but also provides the exact locations of fish fry. Furthermore, it may also be transferred to count other small objects in smart aquaculture.
2. A simple, lightweight and dual branch parallel feature extractor is designed, which can perform multi-scale feature extraction and maintain high-resolution representations. Meanwhile, the computational effort is significantly reduced.
3. Two identical deconvolution modules (TDMs) are added to the generation head, or the keypoint-counting module, to generate a high-quality heatmap that has the same size as the original image with more detailed features. In this way, the proposed method can improve the performance of small object counting in high-density scenes.
4. A large-scale fish fry dataset (FishFry-2023) is constructed. In the dataset, a single point instead of a bounding box is used to mark any individual fish fry when labeling the data, thus significantly reducing the workload of annotating tasks.

## 2. Materials and Methods

### 2.1. Materials

In order to develop an automatic fry counting method, we first constructed a fish fry dataset, namely FishFry-2023. The data in this experiment were collected under the condition that the normal growth of the fish fry was not affected. In the process of data acquisition, we provided an environment suitable for the survival of fish fry to maintain the vitality of fish fry, so as to obtain more abundant sample data.

#### 2.1.1. Fish Fry Images Collection

**Figure 2:**
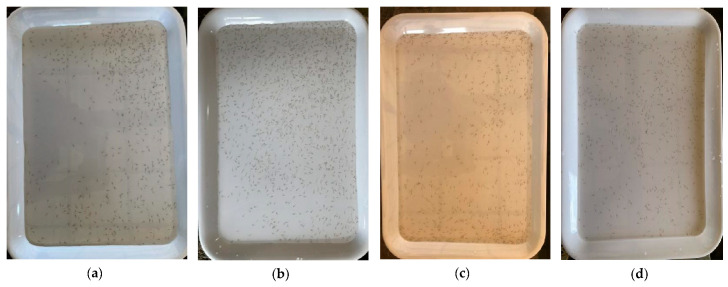

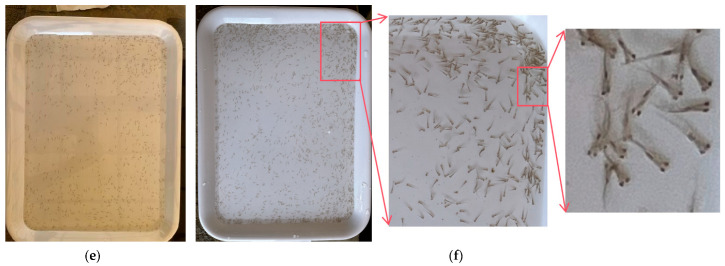
Images with different illumination conditions, watercolors, and levels of density in the FishFry-2023 dataset: (**a**) lightness: reflection; (**b**) water: clear; (**c**) water: slightly muddy; (**d**) density: low level; (**e**) density: medium level; (**f**) density: high level.

We collected the data of this experiment in a farm of fish fry, using equipment such as diddle-nets, white opaque plates, and smartphones, which are readily available and inexpensive. First of all, we used the net to take some fish fry and put them onto the plate filled with water. Then, we took photos of the fry on the plate with our smartphones. Next, after taking a certain number of photographs, we continued to put more fish fry onto the plate for more pictures, repeating this operation to obtain an increasing number of fish fry datasets in a random way. Finally, we acquired 8 batches of images with a total of 1100 images, where the number of fish fry mounted up in batch order, and the number of fish fry images in each batch, was the same. Even though the number of fish fry remains the same in each image of a given batch, the position and the morphology of fish fry in each image are different due to the swimming fish fry. Thus, no images are identical in our counting. The dataset is featured by the diversity, which is specifically demonstrated in the following four aspects:

1. Size variance. Since the smartphone has a local automatic gravity sensing function, the collected images have two different sizes, which are 4032 × 3024 and 3024 × 4032. All images are stored in JPG format without being processed into the same size, and both sizes are used for data labeling and model training.
2. Light variance. Since the data are collected under different lighting conditions, the background of the images is different, including dark light, natural light, and reflection (Figure 2a).
3. Water variance. Since the turbidity of the water differs, the background of the images also varies from clear to slightly muddy (Figure 2b,c).
4. Density variance. When collecting data, we constantly increased the number of fry, so the dataset had three types of density distribution: low, medium and high levels of density. Among them, low density means that the number of fish fry is within the range from 204 to 591, with the corresponding images collected in Batches 1, 2, 3 (Figure 2d). Medium density refers to the number of fry ranging from 642 to 876, with the corresponding images collected in Batches 4, 5, 6 (Figure 2e). High-density indicates that the number of fry ranges between 1312 and 1935, with the corresponding images collected in Batches 7, 8 (Figure 2f).

#### 2.1.2. FishFry-2023 Dataset

There are 8 batches of fish fry data, and the data for batches 1–3 were classified as low density, Batches 4–6 as medium density, and Batches 7–8 as high density. In the first step, we pre-processed the collected data, and removed some blurred images that captured only part of the fish fry on the plate and could not be used to distinguish the fish fry. Subsequently, the graphical image annotation tool Labelme was used for data annotation. Each fish fry in the image was annotated with a point positioned at its head. We selected several images from each batch for annotation and obtained the annotation results of each batch (Table 1). In the first batch, the number of fish fry in each picture was 204, and 45 images were pre-processed altogether, with a total of 9180 fish fry labeled. Finally, 200 annotated images were obtained, with a total target number of 137,390.

To verify the robustness of the model, the training set does not include images of all batches, but the validation set and the test set include images of all batches. First, we selected 40 annotated images from Batches 1, 2, 4 and 7, a total of 160 images to establish a training set, and 5 images from Batches 1 to 8, a total of 40 pictures to form a verification set. The ratio of the training set to the validation set was 8:2. The test set included 800 unlabeled images, with each batch comprising 100 images. This was because the number of fish fry in each batch was the same, and we could calculate the exact number of fish fry in each batch in the test set based on the annotated data. Although there are only 160 images in the training set, the annotated target reaches 103,840. The validation set consists of 40 images, but the number of annotated targets reached up to 33,550. It can be seen that our experimental data are sufficient. Details of the architecture of the FishFry-2023 dataset and the test set are shown in the Appendix A.

### 2.2. Methods

In this section, we first introduce the details of two modules in DEKNet: the feature extraction module and the keypoint-counting module. Then, the process of training and the inference of the model, as well as the evaluation metrics, are interpreted. Finally, the experimental setup is illustrated.

#### 2.2.1. Framework

**Figure 3:**
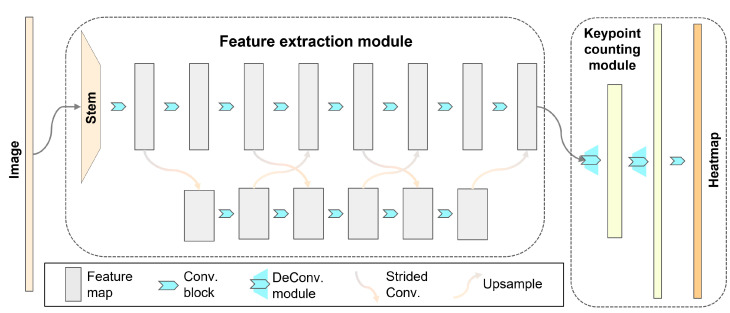
Network architecture of DEKNet. The feature extraction module has two parallel branches to extract target features, which can maintain the resolution of the feature map while performing feature fusion. The keypoint-counting module restores the resolution of the feature map and generates the heatmap.

In this study, we propose a novel counting network that uses only one keypoint and is enhanced by deconvolution, namely DEKNet, for automated, accurate and efficient fish fry counting. In the initial phase, DEKNet is trained on labeled images to obtain its corresponding weights. In a later period, when presented with new images, DEKNet leverages these learned weights to make predictions. During the prediction stage, DEKNet utilizes a heatmap to generate a set of points with coordinate information. Each point corresponds to a detected fish, with the quantity of these points indicating the number of fry. This approach enables DEKNet to accurately count and locate fry within the images. The network framework of DEKNet is composed of two parts, a feature extraction module and a keypoint-counting module inspired, respectively, by HRNet [23] and HigherHRNet. Initially, the feature extraction module extracts target features and outputs feature maps. Subsequently, the keypoint-counting module regresses the keypoint in the form of a heatmap, receives the feature map generated by the backbone, and outputs the keypoint. The schematic diagram of DEKNet is shown in Figure 3.

#### 2.2.2. Feature Extraction Module

Traditional image process methods rely on professionals to manually select object features, resulting in low model robustness. In contrast, deep learning-based models can automatically learn target features through their feature extraction modules, making tasks like image processing more accurately performed. Most existing methods first generate low-resolution representations with rich semantic information through a series of convolution and downsampling modules, and then perform feature fusion and improve the resolution of the feature map through a series of convolution and upsampling modules. For example, feature pyramid networks [24] perform upsampling on the last-layer feature map to improve resolution and perform feature fusion. Improving the resolution of the feature maps may be attributable to the rich spatial information in high-resolution representations, with which object location is facilitated. In our research design, the backbone cannot only obtain more spatial information but also extract high-level semantic features. Therefore, similar to HRNet, our backbone maintains high-resolution representations and fuses multi-scale features throughout the process, rather than recovering high-resolution representations through a low-to-high process. However, in contrast with HRNet, which generates a new branch after each stage of feature fusion due to the downsampling operation, our backbone has only two parallel branches; therefore, it not only allows the model to obtain rich spatial information and semantic features, but also reduces the computational effort of the model.

**Figure 4:**
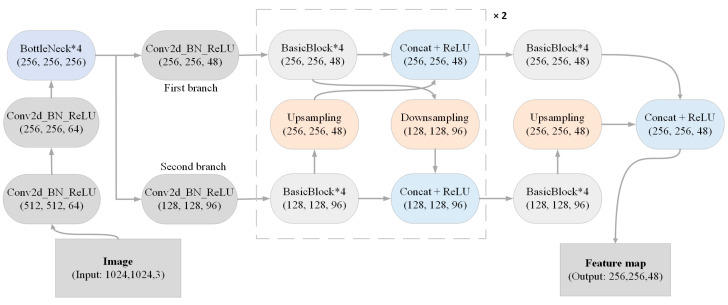
The architecture of the feature extraction module. The input is the image with a resolution of 1024 × 1024. The resolution of the feature map in the first branch is 256 × 256, which is twice that of the second branch. Both branch feature maps require feature fusion operations that only need to be performed twice. Finally, the output is a feature map of 48 channels.

The structure of the feature extraction module is shown in Figure 4. The image feature extraction by backbone consists of the following five steps. (1) The resolution of the image is reduced by two convolution blocks to obtain feature maps whose width and height are one-quarter of the original image. (2) The convolution operation in the BottleNeck module increases the number of channels output, but maintains the resolution of the feature map, and this process is repeated four times. (3) Two branches are generated by two convolution modules. The resolution of the feature map of the first branch remains unchanged, which is used to maintain high-resolution representations. At the same time, the second branch is used to extract high-level semantic features, and the width and height of the feature maps become one-eighth of the original image, and the number of channels in the feature maps is twice that of the first branch. (4) Feature fusion of two branches. First, the two branches extract features through four BasicBlock modules. Next, the first branch is downsampled, and the second branch is upsampled. Eventually, the concat operation is performed on both the feature map in the first branch and the feature map obtained by upsampling in the second branch. Concurrently, the concat operation is performed on both the feature map in the second branch and the feature map obtained by downsampling in the first branch. The same operation is performed twice in this phase. (5) Feature fusion is performed on the first branch only. First, both branches extract features through four BasicBlock modules. Subsequently, the concat operation is performed on the feature map in the first branch, together with the feature map sampled in the second branch to obtain the feature map that can be input to the keypoint-counting module, and the resolution of this feature map is one-quarter of the input image.

#### 2.2.3. Keypoint-Counting Module

A high-resolution heatmap with high quality enables the keypoints of small objects to be localized precisely, so a deconvolution module is added to HigherHRNet to obtain a feature map with the resolution of the input feature map in two folds. Differences between the heads of DEKNet and those of HigherHRNet are found mainly in two aspects. First, we add two identical deconvolution modules to our research. Each deconvolution module can get a new feature map twice the size of the input feature map. Therefore, a high-quality feature map with identical size to the input image is generated by the head. Secondly, only two deconvolution modules are added in our keypoint-counting module, while several other modules are added in HigherHRNet before and after the deconvolution module is used. For example, before the deconvolution module is established, a convolution operation is performed by HigherHRNet to obtain another feature map, and then feature fusion is performed to obtain a new feature map. At the same time, several Basic Residential Blocks [25] are added after the deconvolution module is created.

**Figure 5:**
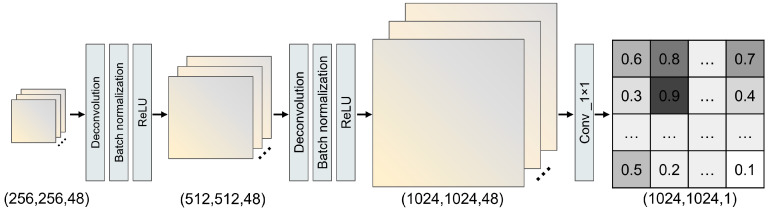
The architecture of the keypoint-counting module. The input is a feature map generated by the feature extraction module, with a resolution of 256 × 256 and a channel of 48. After each deconvolution, the resolution of the feature map is restored twice. Eventually, a heatmap identical in size to the original image is generated.

The architecture of the keypoint-counting module is shown in Figure 5. Two identical deconvolution modules are combined to the head module to generate a high-quality feature map. Eventually, a high-quality prediction heatmap is generated by a convolution operation with core 1 × 1. The workflow of the keypoint-counting module includes three steps. (1) A feature map from backbone is input into the two deconvolution modules to generate a high-quality and high-resolution feature map without changing its channel. (2) The feature map obtained in the previous step is fed into a convolution block to generate one high-quality and high-resolution heatmap for prediction. Because DEKNet only uses a single keypoint for object counting, there is no need to group different types of keypoints, nor is there a need to add additional output. Furthermore, the number of heatmaps and the number of keypoint types have one-to-one correspondence, so the number of channels output by the model is one. (3) Local peaks containing coordinate information in the keypoint heatmap are extracted as the keypoint of the target, and the keypoints are counted to obtain a final result in fish fry counting.

#### 2.2.4. Training and Inference

The heatmap is used to obtain keypoints of the object. During the training, the loss function, defined as mean squared error, is used to calculate the error of the predicted heatmap and the ground truth (GT) heatmap, which is expressed as follows:

(1) $$Loss=\frac{\lambda }{n}\sum_{i=1}^{n}{\left(y_{i}-{\overset{⌢}{y}}_{i}\right)}^{2},$$

where λ is the loss factor, $y_{i}\in \left[0,1\right]$ is the value on the ground truth heatmap, and ${\overset{⌢}{y}}_{i}\in \left[0,1\right]$ is the value on the prediction heatmap.

The inference step represents that the image is input to DEKNet and the counting results are output correspondingly. The pseudo code of inference is shown in Algorithm 1. Firstly, in Algorithm 1, the function conv_some which consists of convolution operation, batch normalization and the activation function is performed on the image to obtain the first feature map (Line 2 in Algorithm 1). The second feature map is obtained by down_sampling operation (Line 3 in Algorithm 1). Then, the function conv_same, which has a function identical to conv_some but maintains the size of the feature map, is performed on the above two feature maps (Lines 5–6 in Algorithm 1). The second feature map only needs to be fused twice, and then the feature fusion operation is performed on the first feature map (Lines 8–11 in Algorithm 1).

Subsequently, the function deconvolution is performed on the first feature map to restore the size of the feature map (Lines 14–16 in Algorithm 1). And the function conv_1 is used to generate the prediction heatmap (Line 17 in Algorithm 1). Lastly, the counting results are obtained by the function obtain_counting_results according to the prediction heatmap (Line 18 in Algorithm 1, see Algorithm 2 for more details).
**Algorithm 1** Pseudo-code of inference**Input:** the original image; window_size; the counting score threshold **Output:** the counting results *CR* 1 preprocess the original results image /* conv_some: a convolution operation; Fmap: feature map */ 2 first_Fmap := conv_some (image) /* create a feature map of the second branch by down_sampling */ 3 second_Fmap := down_sampling (first_Fmap) 4 **for**
*i* in range(0,3) **do** 5  first_Fmap := conv_same (first_Fmap) 6  second_Fmap := conv_same (first_Fmap) 7  upsample_Fmap :=up_sampling (second_Fmap)    /* execute twice to fuse features of the first branch into the second one */ 8  **if** *i* < 2 **then** 9    down_Fmap := down_sampling (first_Fmap) 10    Second_Fmap := concate (second_Fmap, down_Fmap) 11  **end**    /* feature fusion */ 12  first_Fmap := concate (frist_Fmap, upsample_Fmap) 13 **end** /* deconvolution performed twice to get critical features */ 14 **for**
*j* in range (0,2) **do** 15  first_Fmap := deconvolution (first_Fmap) 16 **end** /* conv_1: a convolution operation with core 1×1 */ 17 prediction_heatmap := conv_1 (first_Fmap) /* please refer to Algorithm 2 for the procedure for counting results */ 18 *CR* := obtain_counting_results (prediction_heatmap, window_size, threshold) 19 **return**
*CR*

The heatmap generated by DEKNet can be represented by a two-dimensional array with values between 0 and 1. DEKNet defines a 3 × 3 window to traverse through all the values of the array, keeping the points that match the criteria to represent the number of fry and each point has coordinate information. Relevant details are provided in Algorithm 2. First, the list of counting results CR is initialized (Line 1 in Algorithm 2). Then, the maximum pooling function whose stride is 1 is performed on the heatmap to obtain a temporary heatmap whose size is the same as that of the input heatmap (Line 3 in Algorithm 2). In this work, the kernel size is 3 and the padding is 1. This function enables each value of the heatmap to be replaced with the max value within the kernel size 3 × 3 square region centered at each spatial location (h,w). Subsequently, all the values of the above two heatmaps are iterated. If the two values in the same location are equal, the point is a local peak. At this point, if the value is greater than the score threshold, the coordinate information and the score value of the point are added to the list of counting results CR (Lines 4–11 in Algorithm 2). Finally, the counting result CR is returned (Line 12 in Algorithm 2).
**Algorithm 2** Pseudo-code of obtaining counting results**Input:** the prediction heatmap *PH*; the size of the sliding window;   the counting score threshold **Output:** the counting results *CR* 1 initialization: *CR:= ∅* /* get the height and width of the heatmap */ 2 height, width := *PH*.shape /* each value of the heatmap is the maximum value of this area */ 3 temp_heatmap := MaxPool2d (*PH*, window_size, stride:=1, padding) /* traversing the two heatmaps 4 **for**
*h* in range(height) **do** 5  **for**
*w* in range(width) **do**    /* get the value of the current position */ 6    value := *PH*[h][w]    /* add the location information and value of the keypoint to the *CR* */ 7    **if** value == temp_heatmap[h][w] and value >= threshold **then** 8      CR.append((h, w, value)) 9    **end** 10  **end** 11 **end** 12 **return**
*CR*

#### 2.2.5. Evaluation Metrics

Several performance indicators are utilized to evaluate the performance of the proposed method in our experiments. Accuracy is used to evaluate the counting accuracy of the method. Furthermore, root mean squared error (RMSE) and mean absolute error (MAE) are used to calculate the fish fry counting errors. The specific formulas are as follows:

(2) $$\mathrm{Accuracy}=(\frac{1}{n}\sum_{i=1}^{n}(1-\frac{{|p}_{i}-{\overset{⌢}{p}}_{i}|}{p_{i}}))\times 100\%$$

(3) $$\mathrm{RMSE}=\sqrt{\frac{1}{n}\sum_{i=1}^{n}{(p}_{i}-{\overset{⌢}{p}}_{i}{)}^{2}}$$

(4) $$\mathrm{MAE}=\frac{1}{n}\sum_{i=1}^{n}{|p}_{i}-{\overset{⌢}{p}}_{i}|$$

where n is the total number of images, $p_{i}$ is the ground truth of the i-th image, and ${\overset{⌢}{p}}_{i}$ is the predicted number of the i-th image.

### 2.3. Experimental Setup

The FishFry-2023 dataset used for the training process included a training set of 160 images and a validation set with 40 images, both featuring a resolution of 3024 × 4032 or 4032 × 3024. During the training process, the image was scaled to 1024 × 1024, and the model generated a heatmap of the same size. In addition, to increase the number of training samples and prevent the model from overfitting, the data augmentation techniques employed included horizontal flipping, random scaling ([0.75, 1.5]), and random rotation ([−30°, 30°]). The experiment adopted the hrnet_w48 as pre-trained weights. Furthermore, the Adam optimizer was used. The base learning rate was set to 1.5 × 10^−3^, and dropped to 1.5 × 10^−4^ and 1.5 × 10^−5^ at the 20th and 60th epoch, respectively. The number of training epochs was 500, the batch size was 4, the σ of Gaussian kernels was 1, and the loss factor was 1. The details of the training parameter settings are described in Table 2. In addition to the above hyper parameters, the server equipped with Intel(R) Core i7-11700K CPU, NVIDIA GeForce RTX 3090 GPU (Colorful, Shenzhen, China), PyTorch 1.11.0, CUDA 11.3 and CUDNN 8.2 was used for training and testing.

## 3. Results

In order to validate the performance of the proposed model DEKNet, we designed a series of experiments using different models for comparison. In the first step, we launched experiments on the FishFry-2023 dataset and obtained the results produced by DEKNet. In the second step, to verify the universality of DEKNet, we conducted experiments on the existing Penaeus dataset [26] and a publicly available Adipocyte Cells dataset [27]. Finally, subsequent ablation experiments were carried out on the FishFry-2023 dataset to verify the performance of each component in DEKNet.

### 3.1. Fish Fry Counting Results

As shown in Table 3, DEKNet achieved an accuracy of 98.59% in all images, with the RMSE and MAE being 31.19 and 16.32, respectively. In the low-density level, the counting accuracy was 98.91%, with a RMSE of 7.01 and a MAE of 5.11. In the medium-density level, the counting accuracy was up to 99.05%, with the RMSE and MAE being 13.41 and 7.33, respectively. The counting accuracy in medium density was higher than that of low density, which may be related to the relatively uniform dispersion of fish fry; the more dispersed the fish fry, the more accurate the counting. In the high-density level, the counting accuracy reached 97.44%; its RMSE and MAE were 59.56 and 46.62, respectively. With the increasing number of fish fry in higher levels of dense distribution, the number of overlapping fish fry also increased significantly. As a consequence, the counting accuracy decreased.

**Figure 6:**
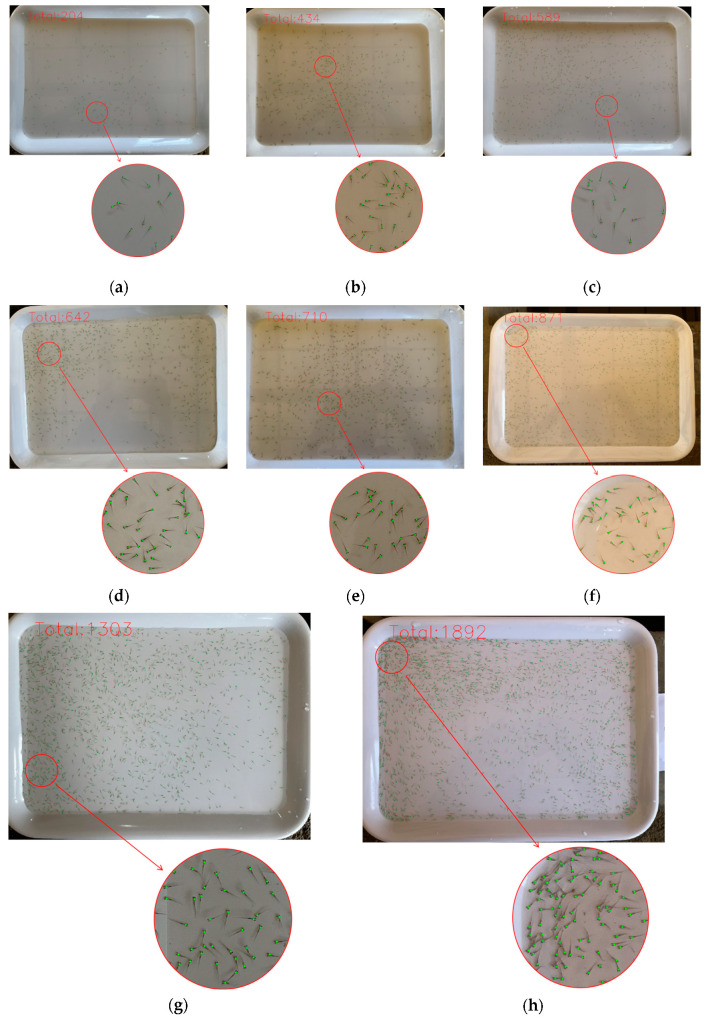
Partial test results of each batch. A green point signifies a fish fry, which is located on the head of any individual fish fry. (**a**) Total number predicted was 204 and GT was 204 in Batch 1; (**b**) total number predicted was 434 and GT was 438 in Batch 2; (**c**) total number predicted was 589 and GT was 591 in Batch 3; (**d**) total number predicted was 642 and GT was 642 in Batch 4; (**e**) total number predicted was 710 and GT was 712 in Batch 5; (**f**) total number predicted was 871 and GT was 876 in Batch 6; (**g**) total number predicted was 1303 and GT was 1312 in Batch 7; and (**h**) total number predicted was 1892 and GT was 1935 in Batch 8.

The partial test results of 8 batches are shown in Figure 6. It can be seen that the size of fish fry is very small; the number of fish fry in each batch is different, and each image has a different background. In Figure 6h, despite the number of fish fry being as large as 1935, they can still be counted individually even in close proximity, which is the advantage of using only one keypoint for counting.

### 3.2. Comparisons with Different Counting Methods

Currently, there are three types of backbone systems. The first type is a typical convolutional neural network (CNN) including HRNet-W48, BL [28], HigherHRNet-W48 and DEKNet, the second type is featured by transformer architecture, including Swin-Base [29] and PVTv2-B2 [30], and the third type combines CNN with transformer architecture, such as HRFormer-S [31]. Among these models, only BL is a density map estimation method, and the other models combine a heatmap to implement the keypoint counting function.

To better display the comprehensive performance of DEKNet, the calculation results of seven methods were compared in each batch of FishFry-2023. The comparison results between DEKNet and the methods concerning the parameter, computational amount and accuracy are shown in Table 4. Many models, such as HRNet [23] and HigherHRNet [21], measure their performance using two indicators: the parameter (Params) and the floating point operations (FLOPs). If a model has fewer Params and FLOPS, it typically indicates that it is lighter in weight and higher in efficiency. Such a model can be deployed and executed more effectively in resource-constrained environments, potentially offering faster inference speeds and lower power consumption. The value in the bold type indicates that a particular method outperforms others concerning a corresponding indicator. (1) The parameters of DEKNet were 3.32 MB which was 4.43 MB less than those of HRFormer-S. Although DEKNet has few parameters, the total computational complexity is still relatively large, as the higher the resolution of the heatmap, the greater the computational complexity of the model. Meanwhile, the FLOPs of DEKNet were 112.24 G, which was 50.34 G more than those of HRFormer-S and remained the third least among the seven methods. When compared to HRNet-W48, the FLOPs of DEKNet decreased by 224.15 G, signifying increased efficiency. (2) DEKNet outperformed the other six models in terms of total average accuracy. For example, compared to BL and HRFormer-S, DEKNet improved average accuracy by 0.75 and 2.87 points, respectively. (3) DEKNet and BL were very close to each other in accuracy from Batch 1 to Batch 5, all exceeding 98%. In Batch 2, the accuracy of BL was 98.83, only 0.02% higher compared with DEKNet. Despite this, as the number of fish fry increased, the accuracy of DEKNet was significantly higher than that of BL. (4) The accuracy of DEKNet in Batches 7–8 was 99.08% and 95.80%, respectively, 2.18% and 1.19% higher than that of BL. By contrast, all the other five methods had an accuracy rate of less than 87% in Batches 7–8. For example, the accuracy of PVTv2-B2 was the lowest at 72.85%. In addition, the accuracy of HRNet-W48 in the 8th batch was only 80.08%, 15.72% less than that of DEKNet.

Furthermore, the fish fry counting errors in each model, namely RMSE and MAE, are shown in Table 5. Since the number of fish fry in each image in the first batch was only 204, the RMSE and MAE of all methods were relatively small. For example, the RMSE and MAE of HRNet were 11.69 and 11.19, respectively, and those of DEKNet were 1.55 and 1.12, respectively. It can be seen that the RMSE and MAE were much lower in DEKNet with the exception of the second batch; the RMSE and MAE of DEKNet were significantly lower than those of the other six methods. In the second batch, the RMSE and MAE of BL were 1.6 and 1.1 less than those of DEKNet, respectively. In addition, the RMSE of DEKNet in the 7th and 8th batches was 14.40 and 82.99, respectively, which was lower than that of the other six methods. Meanwhile, in the 8th batch, the MAE of DEKNet was 81.19, 177.01 lower than that of HigherHRNet, 217.82 lower than that of Swin, and 187.01 lower than that of HRFormer.

**Figure 7:**
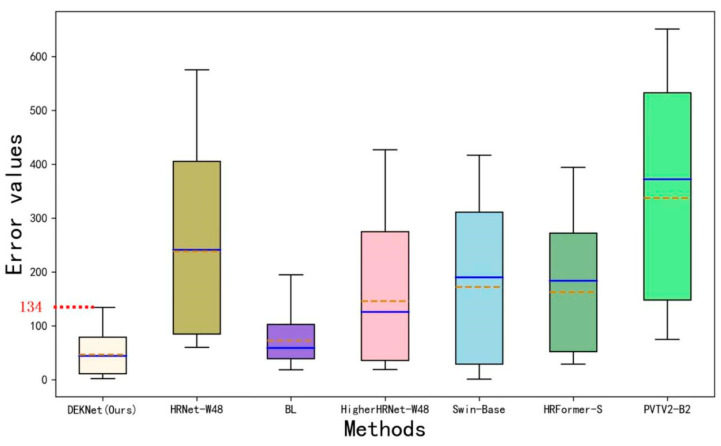
Box plots of error values between the predicted values and the ground truth for seven different methods.

**Figure 8:**
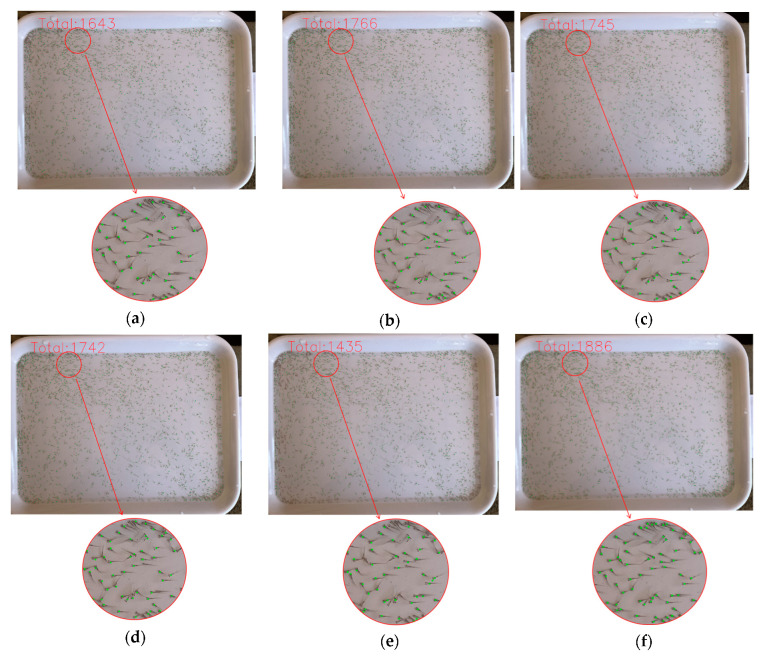
Comparisons of the total number of fish fry predicted by six different methods in the same image whose GT was 1935. (**a**) The total number predicted by HRNet-W48 was 1643; (**b**) the total number predicted by HigherHRNet-W48 was 1766; (**c**) the total number predicted by Swin-Base was 1745; (**d**) the total number predicted by HRFormer-S was 1742; (**e**) the total number predicted by PVTV2-B2 was 1435; and (**f**) the total number predicted by DEKNet (ours) was 1886.

In addition, to more intuitively demonstrate the counting performance of DEKNet at high-density levels, we plotted box plots of the error values between the predicted values and the ground truth for the above models. As shown in Figure 7, the orange line represents the mean value, and the blue line represents the median value. The minimum value and the maximum value of errors are, respectively, represented by two short lines outside the box. It can be seen that the max error value of DEKNet was 134, which was smaller than that of the other six methods. Furthermore, DEKNet has the lowest box height, which manifests the best stability and the least data fluctuation. What is more, as shown in Figure 8, the total number of fish fry predicted by DEKNet was 1888 and the accuracy was 97.57%, which was obviously superior to the other models in the same sample. As BL could not predict the locations of fish fry, we did not show the image predicted by BL in Figure 8.

In summary, DEKNet was more robust and accurate in counting fish fry compared with the other six methods. In Batch 7 and Batch 8, marked by particularly dense distribution, the comprehensive performance of DEKNet was more satisfactory than the other six methods in the comparison. The reason why DEKNet is excellent mainly lies in the two deconvolution modules added to the generation head for a high-resolution and high-quality heatmap.

### 3.3. Comparisons with Crowd-Counting Methods

A series of experiments were conducted on FishFry-2023 by employing state-of-the-art crowd-counting methods such as CSRNet [32], CAN [33], CCTrans [34], SCALNet [35], and P2PNet [36]. The comparison results on the test set are presented in Table 6. In terms of parameters and FLOPs, DEKNet surpassed these crowd-counting methods. With the exception of DEKNet and SCALNet, all the other methods exhibited FLOPs exceeding 300 G. While SCALNet boasted the second smallest FLOPs, it still exceeded DEKNet’s by 31.27 G. This demonstrated that DEKNet was more efficient than these crowd-counting methods. Furthermore, our method exhibited superior performance according to the evaluation metrics, closely followed by P2PNet. The accuracy for CSRNet and CAN was 81.10% and 84.58%, respectively, whereas the accuracy for CCTrans, SCALNet, and P2PNet all exceeded 90%. In comparison to CCTrans, our method demonstrated an improvement in accuracy (increased by 2.53%). Furthermore, compared to P2PNet, our method achieved an increase in accuracy (improved by 2.01%), accompanied by reductions of 16.46 and 16.1 in RMSE and MAE, respectively. The modest performance of these crowd-counting methods for fish fry counting may be attributed to the diminutive size of the fry, which contrasts sharply with the larger size of humans. These findings underscore the efficacy of a high-resolution heatmap in facilitating small object counting, thereby positioning the proposed method favorably relative to certain crowd-counting methodologies.

### 3.4. Comparisons Results on Penaeus Dataset

To validate the generalization ability of DEKNet, we conducted experiments on a Penaeus dataset using the models introduced in Section 3.2. The Penaeus dataset is a shrimp fry dataset of Penaeus monodon, which consists of a training set with 593 images, a validation set with 60 images and a test set with 1426 images.

The results on the test set are shown in Table 7. Compared to HRNet-W48 [23] and BL [28], DEKNet improved average accuracy by 7.07 and 2.55 points, respectively, in all test images. At the same time, the RMSE of DEKNet was 25.68, 101.22 less than that of HRNet-W48, and 30.32 less than that of BL. What is more, the MAE of DEKNet was 14.77, which was less than that of the other six methods. In all test images, compared to Swin-Base [29] and HRFormer-S [31], our method achieved a 8.21 and 6.66 point gain in accuracy, respectively. Furthermore, the accuracy and the MAE of PVTv2-B2 [30] were 92.25% and 34.59, respectively, which were less than those of DEKNet.

**Figure 9:**
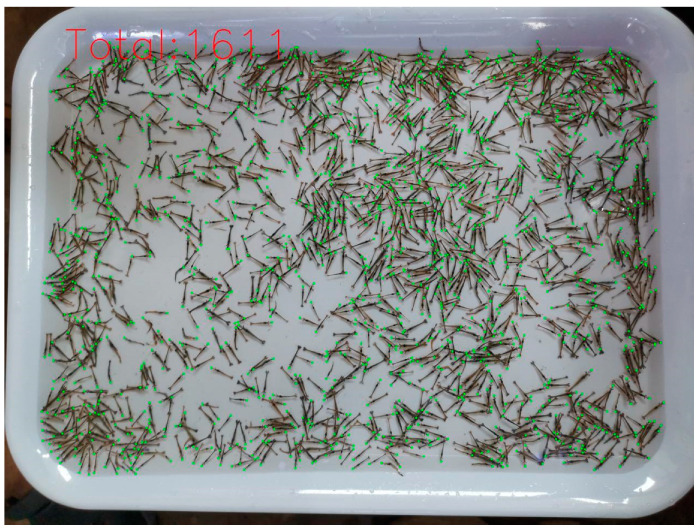
An example image of the Penaeus dataset whose ground truth is 1691. The total number predicted by DEKNet was 1611. A green point represents a penaeus monodon, which is located on the head of individual shrimp fry.

The test result in one of the images with the largest number of shrimp fry is shown in Figure 9. Although the shrimp fry were very densely distributed and severely occluded, our method still made efficient counting by means of the keypoint of the head, with an accuracy reaching 95.27%, and its predicted value and ground truth were 1611 and 1691, respectively. In summary, our method also outperformed the other six methods in the Penaeus dataset. The foremost reason is the fact that DEKNet can generate a heatmap to precisely locate the keypoint of small objects.

### 3.5. Comparisons Results on Adipocyte Cells Dataset

In addition, we made comparisons between DEKNet and two cell-counting methods, namely Count-ception [37] and SAU-Net [38]. The Adipocyte Cells dataset consists of 200 images. The average cell count across all images is 165 ± 44.2 and they are densely packed, adjoining cells with few gaps [37]. Based on the experimental settings of these two cell-counting methods, we randomly selected 50 images for the training set, 50 images for the validation set, and the remaining 100 images for the test set every time. This process was conducted at least 10 times, and eventually the mean value (MAE) and its standard deviation (STD) were given. The MAE and STD of these two methods came from their respective experimental outcomes. The DEKNet reduced the MAE by 6.1 and STD by 1, respectively, compared to Count-ception. Compared with SAU-Net, the MAE and STD of our method decreased by 0.88 and 0.4, respectively. The experimental results are shown in Table 8.

All in all, our method outperformed the above methods in the public dataset of the Adipocyte Cells dataset. The superior performance of DEKNet can be attributed to two factors, namely our method cannot only maintain the high representations, but also generate a high-resolution heatmap with high quality.

### 3.6. Ablative Analysis of Each Module

In order to study the effectiveness of each component, we conducted ablation experiments in the test set of FishFry-2023. The experimental results are shown in Table 9. Let H and W represent the height and width of the input image, respectively. With only our fish feature extractor (FFE) and no deconvolution modules in the generation head, Experiment A generated a heatmap size of H/4 × W/4 with a counting accuracy of 88.87%. And the RMSE and MAE of Experiment A were 202.89 and 120.80, respectively.

Experiment B and Experiment C were conducted on the basis of Experiment A. In both experiments, one deconvolution module (ODM) was added, but the size of the heatmap they generated was different because the stride of the ODM was different. ODM1 represents that the stride is 2 and ODM2 represents that the stride is 4. The size of the heatmap generated by Experiment B is H/2 × W/2, while that generated by Experiment C is H × W. Compared with Experiment A, the counting accuracy of Experiment B increased by 8.48%, and its RMSE and MAE decreased to 64.72 and 33.50, respectively. This indicates that improving the resolution of the heatmap will achieve a higher calculation accuracy of the model. Compared with Experiment B, all indicators in Experiment C were better than those in Experiment B.

Experiment D was performed by DEKNet, where a deconvolution module was added based on Experiment B. Experiment D involved two identical deconvolution modules with the same stride, generating a heatmap with a size of H × W. Compared with the previous experiments, the indicators in Experiment D are optimal. The accuracy of experiment D was 98.59%, up by 1.24% compared with Experiment B and up by 0.7% compared with Experiment C. The RMSE and MAE of Experiment D were 31.19 and 16.32, respectively, which were 33.53 and 17.18 less than those in Experiment B, and 16.25 and 4.82 less than those in Experiment C.

The above ablation experiments show that each component is effective, and the high-resolution heatmap with high quality is conducive to precisely locating the keypoint of small objects. Therefore, our method is optimal; adding two individual deconvolution modules to the generation head is better than just adding one. Similarly, adding two deconvolution modules is better than adding only one, provided that the heatmap resolution is restored to the input image resolution.

### 3.7. Ablative Analysis of Different Image Sizes

We maintained the remaining parameters constant and conducted a series of experiments by resizing the original image to various dimensions. The outcomes are summarized in Table 10. Experiment A utilized an image size of 256 × 256, yielding an accuracy rate of merely 33.60%. Across Experiments A to E, the counting accuracy of DEKNet consistently improved with increasing image dimensions. Nevertheless, in Experiment F, the accuracy declined, reaching only 96.60%. These experiments highlight the impact of image size on DEKNet’s counting performance—undersized images hinder accuracy, while excessively large images are also suboptimal. Thus, we opted to resize the image to 1024 × 1024 for optimal performance.

In the Results section, a series of experiments were conducted to assess the counting performance of DEKNet. Initially, the counting results of DEKNet in FishFry-2023 were presented, alongside a comparative analysis with the keypoint-counting methods and the crowd-counting methods. Subsequently, to evaluate DEKNet’s generalization capabilities, a set of comparative experiments was performed on two additional datasets, namely the Penaeus dataset and the Adipocyte Cells dataset. Finally, two sets of ablation experiments were introduced; one was introduced to verify the effect of each module of DEKNet, the other to explain the rationale behind setting the image size to 1024 × 1024. The experimental findings demonstrated that DEKNet outperformed the methods in the comparative experiments in terms of parameter, FLOPs, and counting accuracy.

## 4. Discussion

The low accuracy observed in existing counting methods can largely be attributed to factors such as the small size, high density, and the mutual occlusion of fish fry. In order to address these challenges and enhance fry-counting precision, a novel methodology utilizing a singular keypoint is proposed within this study. Presently, there are limited methods for fry counting utilizing keypoints, and research on generating a high-resolution heatmap is scarce [21]. DEKNet’s production of a high-resolution heatmap addresses this gap, realizing precise localization of small targets and minimizing errors. Consequently, when the heatmap resolution matches that of the input image, the localization accuracy improves significantly, resulting in fewer errors [20]. Furthermore, DEKNet can be used to count the penaeus [26] and the cells [27]. Therefore, DEKNet is more robust compared to existing methods in the research field of fish fry counting. The exemplary performance of DEKNet can be primarily ascribed to the integration of two identical deconvolution modules within its keypoint-counting module, thereby advancing the generation of a high-resolution and high-quality heatmap. Nevertheless, it is imperative to acknowledge that the higher-resolution heatmap necessitates increased computational resources. Under the comprehensive trade-off, DEKNet strategically incorporates a lightweight feature extraction module, thereby attaining high-resolution representations while minimizing computational overhead.

Our research still has some limitations. Since DEKNet only uses a keypoint to identify targets, it is only suitable for counting targets of the same category, and lacks the ability to separately and synchronously count the targets of different categories in the same image, which is one of its shortcomings. Another limitation of DEKNet is that it requires that selected keypoint to uniquely identify an object; if the keypoints in multiple positions of the object are indistinguishable, the counting effect of DEKNet will be reduced. For example, if a fish has two eyes, assuming that most of the characteristics of these two eyes resemble each other, and that only the left eye is selected as a keypoint, then DEKNet may also count its right eye together with its left eye, which will increase the error of fry counting. Therefore, DEKNet’s selection of the object keypoint is an important factor affecting its counting performance.

In the future, we will endeavor to optimize DEKNet so that it cannot only accurately count fish fry with different day age, but also deal with large quantities of them at the same time. Furthermore, DEKNet can be trained to recognize multiple keypoints of different categories, and then fulfill the function of counting different targets simultaneously. Hopefully, we will apply it to other research subjects in intelligent agriculture, such as counting poultry, legumes, and aquatic products.

## 5. Conclusions

In this work, a keypoint-based counting network, DEKNet, is proposed and a large-scale dataset (FishFry-2023) is constructed. Drawing inspiration from the architectural principles of HRNet and HigherHRNet, the framework of DEKNet is established. Comparative analysis demonstrates that DEKNet surpasses alternative keypoint-counting methods in terms of accuracy rates. Furthermore, DEKNet enables accurate fry localization. The backbone of DEKNet maintains high-resolution representations throughout the process and uses only two parallel branches to extract target features, which can significantly reduce the model size. Furthermore, two identical deconvolution modules added to the generation head are conducive to generating an accurate and spatially precise keypoint heatmap with high quality and high resolution. The experimental results indicate that the accuracy of DEKNet reached 98.59%, with an RMSE of 31.19 and an MAE of 16.32 in the FishFry-2023 dataset. In summary, DEKNet can be trained for direct prediction on the complete image, thus avoiding repeated counting of the boundary area when the image is segmented into several sub-images. What is more, this model stands out in counting fish fry and even smaller objects.

## Figures and Tables

**Table 1:** Annotation results of each batch.

Density	Low Level			Medium Level			High Level	
Batch number	1	2	3	4	5	6	7	8
Image	45	45	5	45	5	5	45	5
Fry number in per image	204	438	591	642	712	876	1312	1935
Instance	9180	19,710	2955	28,890	3560	4380	59,040	9675

**Table 2:** Training parameter setting.

Parameter	Value
Input size	1024 × 1024
Pre-trained weight	hrnet_w48
Epoch	500
Optimizer	Adam
Learning rate	1.5 × 10^−3^
Batch size	4
σ	1
λ	1

**Table 3:** Experimental results of fish fry counting at different levels of density.

Density Level	Accuracy (%)	RMSE	MAE
Low	98.91	7.01	5.11
Medium	99.05	13.41	7.33
High	97.44	59.56	46.62
Total	98.59	31.19	16.32

**Table 4:** Experimental results in low-, medium- and high-density fish fry. The value in the bold type indicates that a particular method outperforms others concerning a corresponding indicator.

Method	Year	Params (MB)	FLOPs (G)	Accuracy ↑ (%)								
				1	2	3	4	5	6	7	8	Total
HRNet-W48	2019	63.59	336.39	94.51	88.73	94.06	94.54	95.50	83.77	93.04	80.08	90.53
BL	2019	21.50	421.73	99.28	**98.83**	98.34	98.27	99.17	97.29	96.90	94.61	97.84
HigherHRNet-W48	2020	63.80	382.75	96.66	94.85	95.54	95.65	98.43	94.73	96.94	86.98	94.97
Swin-Base	2021	86.75	334.19	91.92	81.70	95.01	79.89	88.25	79.84	97.91	84.57	89.26
HRFormer-S	2021	7.75	**61.90**	99.01	97.65	97.69	97.09	98.79	93.76	96.61	86.16	95.72
PVTv2-B2	2022	24.85	68.35	96.30	91.63	94.87	81.70	83.96	67.87	88.60	72.85	84.72
DEKNet (ours)	2023	**3.32**	112.24	**99.45**	98.79	**98.49**	**98.83**	**99.77**	**98.53**	**99.08**	**95.80**	**98.59**

**Table 5:** Comparisons of RMSE and MAE among different methods. The value in the bold type indicates that a particular method outperforms others concerning a corresponding indicator.

Method	1		2		3		4		5		6		7		8	
	RMSE	MAE	RMSE	MAE	RMSE	MAE	RMSE	MAE	RMSE	MAE	RMSE	MAE	RMSE	MAE	RMSE	MAE
HRNet	11.69	11.19	52.50	49.33	36.10	35.11	41.27	35.01	33.97	32.04	231.68	142.20	96.42	91.25	394.16	385.46
BL	1.67	1.47	**5.54**	**5.11**	10.64	9.80	12.44	11.12	6.51	5.89	25.32	23.75	42.37	40.73	108.06	104.31
HigherHRNet	7.06	6.82	23.71	22.54	28.12	26.38	36.60	27.93	11.97	11.20	66.10	46.15	44.60	40.10	260.83	251.91
Swin	18.40	16.48	98.70	80.16	33.47	29.47	172.85	129.12	119.13	83.69	328.12	185.4	40.75	27.43	300.81	298.58
HRFormer	2.42	2.02	10.79	10.30	15.83	13.63	23.02	18.67	9.49	8.58	76.41	54.70	63.32	57.60	270.20	267.85
PVTv2	10.37	7.54	48.46	36.65	32.48	30.33	137.94	117.55	127.07	114.23	362.75	281.42	155.17	149.54	526.89	525.31
DEKNet	**1.55**	**1.12**	5.94	5.26	**10.47**	**8.94**	**11.96**	**7.51**	**2.12**	**1.63**	**19.79**	**12.84**	**14.40**	**12.04**	**82.99**	**81.19**

**Table 6:** Comparisons results on FishFry-2023 test set. The value in the bold type indicates that a particular method outperforms others concerning a corresponding indicator.

Method	Venue	Params (MB)	FLOPs (G)	Accuracy (%)	RMSE	MAE
CSRNet	CVPR’18	16.26	433.79	81.1	225.53	157.71
CAN	CVPR’19	18.1	459.72	84.58	207.78	128.28
CCTrans	CVPR’21	104.61	397.37	90.61	73.14	55.73
SCALNet	IEEE’21	19.1	143.51	94.05	139.98	62.44
P2PNet	ICCV’21	21.58	383.31	96.58	47.65	32.42
DEKNet	-	**3.32**	**112.24**	**98.59**	**31.19**	**16.32**

**Table 7:** Comparisons of the Acc, RMSE and MAE of different methods on the Penaeus dataset. The value in the bold type indicates that a particular method outperforms others concerning a corresponding indicator.

Method	Accuracy(%)	RMSE	MAE
HRNet-W48	91.44	126.90	69.20
BL	95.96	56.60	27.72
HigherHRNet-W48	95.66	65.75	36.59
Swin-Base	90.30	120.02	57.86
HRFormer-S	91.85	113.16	64.96
PVTv2-B2	92.25	49.26	34.59
DEKNet (ours)	**98.51**	**25.68**	**14.77**

**Table 8:** Comparison results between our method and two cell-counting methods on Adipocyte Cells. The value in the bold type indicates that a particular method outperforms others concerning a corresponding indicator.

Method	MAE ± STD
Count-ception	19.40 ± 2.2
SAU-Net	14.20 ± 1.6
DEKNet (ours)	**13.32 ± 1.2**

**Table 9:** Ablation study of each module on FishFry-2023 test dataset.

Experiment	Method	Number of Deconvolution Module	Heatmap Size	Accuracy (%)	RMSE	MAE
A	FFE	0	H/4 × W/4	88.87	202.89	120.80
B	FFE + ODM1	1	H/2 × W/2	97.35	64.72	33.50
C	FFE + ODM2	1	H × W	97.89	47.44	21.14
D	FFE + TDM(DEKNet)	2	H × W	98.59	31.19	16.32

**Table 10:** Ablation study of different image sizes.

Experiments	Image_Size	Accuracy (%)
A	256 × 256	33.60
B	640 × 640	44.94
C	720 × 720	45.83
D	1000 × 1000	89.96
E	1024 × 1024	98.51
F	1400 × 1400	96.60

## Data Availability

The FishFry-2023 datasets presented in this study can be accessed at the following link: [https://pan.baidu.com/s/1HyOgpMiQrQQhBwV_imn81A] (accessed on 1 May 2024) and the code is 2qvu.

## Appendix A

The appendix is about the detailed architecture of FishFry-2023.

**Table A1:** Dataset structure of FishFry-2023.

Dataset	Image	Instance	Annotation Point	Batch Number
Train set	160	103,880	103,880	1, 2, 4, 7
Validation set	40	33,570	33,570	1–8
Test set	800	670,900	-	1–8

**Table A2:** Details of FishFry-2023 test set.

Density Level	Batch Number	Image	Range
Low	1, 2, 3	300	204–592
Medium	4, 5, 6	300	642–876
High	7, 8	200	1312–1935
Total	1–8	800	204–1935

## References

[1] Liu S., Zeng X., Whitty M. (2020). A vision-based robust grape berry counting algorithm for fast calibration-free bunch weight estimation in the field. Comput. Electron. Agric..

[2] Xu C., Jiang H., Yuen P., Ahmad K.Z., Chen Y. (2020). MHW-PD: A robust rice panicles counting algorithm based on deep learning and multi-scale hybrid window. Comput. Electron. Agric..

[3] Han T., Bai L., Gao J., Wang Q., Ouyang W.L. Dr. Vic: Decomposition and reasoning for video individual counting. Proceedings of the IEEE/CVF Conference on Computer Vision and Pattern Recognition.

[4] Akçay H.G., Kabasakal B., Aksu D., Demir N., Öz M.E.A. (2020). Automated bird counting with deep learning for regional bird distribution mapping. Animals.

[5] Fan L., Liu Y. (2013). Automate fry counting using computer vision and multi-class least squares support vector machine. Aquaculture.

[6] Zhang J., Pang H., Cai W., Yan Z. (2022). Using image processing technology to create a novel fry counting algorithm. Aquac. Fish..

[7] Hernández-Ontiveros J.M., Inzunza-González E., García-Guerrero E.E., López-Bonilla O.R., Infante-Prieto S.O., Cárdenas-Valdez J.R., Tlelo-Cuautle E. (2018). Development and implementation of a fish counter by using an embedded system. Comput. Electron. Agric..

[8] Aliyu I., Gana K.J., Musa A.A., Adegboye M.A., Lim C.G. (2020). Incorporating recognition in catfish counting algorithm using artificial neural network and geometry. Ksii. T. Internet. Inf..

[9] Zhang L., Li W., Liu C., Zhou X., Duan Q. (2020). Automatic fish counting method using image density grading and local regression. Comput. Electron. Agric..

[10] Zhao Y., Li W., Li Y., Qi Y., Li Z., Yue J. (2022). LFCNet: A lightweight fish counting model based on density map regression. Comput. Electron. Agric..

[11] Yu C., Hu Z., Han B., Dai Y., Zhao Y., Deng Y. (2023). An intelligent measurement scheme for basic characters of fish in smart aquaculture. Comput. Electron. Agric..

[12] Ditria E.M., Lopez-Marcano S., Sievers M., Jinks E.L., Brown C.J., Connolly R.M. (2020). Automating the analysis of fish abundance using object detection: Optimizing animal ecology with deep learning. Front. Mar. Sci..

[13] Allken V., Rosen S., Handegard N.O., Malde K. (2021). A deep learning-based method to identify and count pelagic and mesopelagic fishes from trawl camera images. Ices. J. Mar. Sci..

[14] Jalal A., Salman A., Mian A., Shortis M., Shafait F. (2020). Fish detection and species classification in underwater environments using deep learning with temporal information. Ecol. Inform..

[15] Cai K., Miao X., Wang W., Pang H., Liu Y., Song J. (2020). A modified YOLOv3 model for fish detection based on MobileNetv1 as backbone. Aquac. Eng..

[16] Lei J., Gao S., Rasool M.A., Fan R., Jia Y., Lei G. (2023). Optimized small waterbird detection method using surveillance videos based on YOLOv7. Animals.

[17] Zhou X., Wang D., Krähenbühl P. (2019). Objects as points. arXiv.

[18] Liu G., Hou Z., Liu H., Liu J., Zhao W., Li K. (2022). TomatoDet: Anchor-free detector for tomato detection. Front. Plant. Sci..

[19] Chen G., Shen S., Wen L., Luo S., Bo L. (2020). Efficient pig counting in crowds with keypoints tracking and spatial-aware temporal response filtering. arXiv.

[20] Nibali A., He Z., Morgan S., Prendergast L. (2008). Numerical coordinate regression with convolutional neural networks. arXiv.

[21] Cheng B., Xiao B., Wang J., Shi H., Huang T.S., Zhang L. (2020). HigherHRNet: Scale-Aware representation learning for Bottom-Up human pose estimation. arXiv.

[22] Xiao B., Wu H., Wei Y. Simple baselines for human pose estimation and tracking. Proceedings of the European Conference on Computer Vision.

[23] Sun K., Xiao B., Liu D., Wang J. (2019). Deep High-Resolution representation learning for human pose estimation. arXiv.

[24] Lin T., Dollár P., Girshick R., He K., Hariharan B., Belongie S. (2017). Feature pyramid networks for object detection. arXiv.

[25] He K., Zhang X., Ren S., Sun J. (2015). Deep residual learning for image recognition. arXiv.

[26] Li X., Liu R., Wang Z., Zheng G., Lv J., Fan L., Guo Y.B., Gao Y.F. (2023). Automatic penaeus monodon larvae counting via equal keypoint regression with smartphones. Animals.

[27] Lonsdale J., Thomas J., Salvatore M., Phillips R., Lo E., Shad S. (2013). The genotype-tissue expression (GTEx) project. Nat. Genet..

[28] Ma Z., Wei X., Hong X., Gong Y. Bayesian loss for crowd count estimation with point supervision. Proceedings of the IEEE/CVF International Conference on Computer Vision (ICCV).

[29] Liu Z., Lin Y., Cao Y., Hu H., Wei Y., Zhang Z., Lin S., Guo B.N. Swin transformer: Hierarchical vision transformer using shifted windows. Proceedings of the IEEE/CVF International Conference on Computer Vision (ICCV).

[30] Wang W., Xie E., Li X., Fan D., Song K., Liang D., Lu T., Luo P., Shao L. (2022). Pvt v2: Improved baselines with pyramid vision transformer. Comput. Vis. Media.

[31] Yuan Y., Fu R., Huang L., Lin W., Zhang C., Chen X., Wang J.D. (2021). HRFormer: High-Resolution transformer for dense prediction. arXiv.

[32] Li Y.C. Dilated convolutional neural networks for understanding the highly congested scenes. Proceedings of the IEEE Conference on Computer Vision and Pattern Recognition.

[33] Liu W., Salzmann M., Fua P. Context-aware crowd counting. Proceedings of the IEEE/CVF Conference on Computer Vision and Pattern Recognition.

[34] Tian Y., Chu X., Wang H. (2021). CCTrans: Simplifying and improving crowd counting with transformer. arXiv.

[35] Wang Y., Hou X., Chau L. (2021). Dense point prediction: A simple baseline for crowd counting and localization. arXiv.

[36] Song Q., Wang C., Jiang Z., Wang Y., Tai Y., Wang C., Li J., Huang F., Wu Y. (2021). Rethinking counting and localization in crowds:a purely Point-Based framework. arXiv.

[37] Cohen J.P., Boucher G., Glastonbury C.A., Lo H.Z., Bengio Y. (2017). Count-ception: Counting by fully convolutional redundant counting. arXiv.

[38] Guo Y., Stein J., Wu G., Krishnamurthy A. SAU-Net: A universal deep network for cell counting. Proceedings of the 10th ACM International Conference on Bioinformatics, Computational Biology and Health Informatics.

